# Centromere Chromatin Dynamics at a Glance

**DOI:** 10.3390/epigenomes6040039

**Published:** 2022-11-03

**Authors:** Shivangi Shukla, Ashutosh Kumar

**Affiliations:** NMR-Based Structural Biology Lab, Lab No. 606, Department of Bioscience and Bioengineering, Indian Institute of Technology, Mumbai 400076, India

**Keywords:** CENP-A, H2A.Z, centromere, heterochromatin, kinetochore, PTM

## Abstract

The centromere is a specialized DNA locus that ensures the faithful segregation of chromosomes during cell division. It does so by directing the assembly of an essential proteinaceous structure called the kinetochore. The centromere identity is primarily epigenetically defined by a nucleosome containing an H3 variant called CENP-A as well as by the interplay of several factors such as differential chromatin organization driven by CENP-A and H2A.Z, centromere-associated proteins, and post-translational modifications. At the centromere, CENP-A is not just a driving force for kinetochore assembly but also modifies the structural and dynamic properties of the centromeric chromatin, resulting in a distinctive chromatin organization. An additional level of regulation of the centromeric chromatin conformation is provided by post-translational modifications of the histones in the CENP-A nucleosomes. Further, H2A.Z is present in the regions flanking the centromere for heterochromatinization. In this review, we focus on the above-mentioned factors to describe how they contribute to the organization of the centromeric chromatin: CENP-A at the core centromere, post-translational modifications that decorate CENP-A, and the variant H2A.Z.

## 1. Introduction

Cell division aims at proper chromosome segregation to daughter cells such that a complete and accurate copy of the genome is faithfully transmitted to the next generation. To ensure this faithful segregation, a specialized structure called the ‘centromere’ is present at a specific locus of the chromosome. Walther Flemming first described the centromere as ‘primary constrictions’ that are present on condensed chromosomes [1]. Electron microscopy later revealed that the centromere is a chromosomal locus that forms the platform for the assembly of the kinetochore (KT)—a proteinaceous complex that attaches the sister chromatids to the spindle microtubules at the metaphase [2]. Failure of centromere function during mitosis can lead to aneuploidy which can result in genetic instability and predisposition to diseases such as cancer [3] whereas failure during meiosis can lead to developmental defects [4]. In higher eukaryotes such as humans, the centromere sequence is highly repetitive with a high sequence variability (called α-satellite), indicating that the DNA sequence has little role to play in establishing the centromere identity [5]. Further, the formation of functional ‘neocentromeres’ at ectopic locations [6,7], confirms that the DNA sequence is not the major determinant in defining the centromere. Various studies have demonstrated that it is the H3 variant protein-CENP-A, that is the primary determinant of centromere identity [8,9,10,11]. Other factors include Post-translational modifications (PTMs) of histones in CENP-A nucleosomes, centromere-associated proteins, the presence of other histone variants such as H2A.Z, and differential chromatin organization [8]. The centromeric chromatin is made up of the core centromeric region that has CENP-A nucleosomes interspersed with H3 nucleosomes [12] and the pericentromeric heterochromatin. Both regions work in association to form a functional centromere. In this minireview, we describe a few factors that define the centromere. Specifically, we highlight the unique characteristics of the human CENP-A and how they support the establishment and maintenance of the centromere: the PTMs on CENP-A that aid in the former, and the H2A.Z variant that is predominantly present at the pericentromere.

## 2. The Centromere

Lower eukaryotes, such as *Saccharomyces cerevisiae* (budding yeast), have a conserved centromeric DNA sequence, whereas, the centromere sequence in higher eukaryotes are highly variable. The sequence of the centromeric DNA is neither necessary nor sufficient for the formation of the kinetochore [13]. The identity of the centromere is rather determined epigenetically by the incorporation a of Histone H3 variant (CenH3) called Cse4 in *S. cerevisiae* (budding yeast), and CENP-A in humans in the centromeric nucleosome(s) now called the specialized nucleosome(s) [14,15]. Two types of centromeres have been identified in eukaryotes: point and regional. Point centromeres are defined by the presence of a conserved DNA sequence and a single CenH3-containing nucleosome as in the case of the budding yeast [16] while regional centromeres are much more complex and made up of multiple copies of repetitive DNA (heterochromatin) which can be in the order of megabases and contain multiple CenH3 nucleosomes such as in humans [17,18].

Interestingly, the repetitive DNA sequences (called alpha satellite in humans) have little role in specifying the centromere identity as a formation of ‘neocentromeres’ have been observed at extra-centromeric genome sites where CENP-A is incorporated followed by the establishment of a functional kinetochore [19]. Additionally, the loss of CENP-A leads to failure of centromere formation and kinetochore assembly, resulting in mitotic arrest or embryogenic death [20,21]. Here, CENP-A is solely responsible for maintaining and propagating the centromere identity. Despite the structural divergence, the function of CenH3 is highly conserved as CENP-A loss of function can be compensated by Cse4, suggesting that the fundamental features of the centromeric chromatin are conserved across yeast and mammals [22].

## 3. Centromere Protein A (CENP-A)

**Figure 1:**
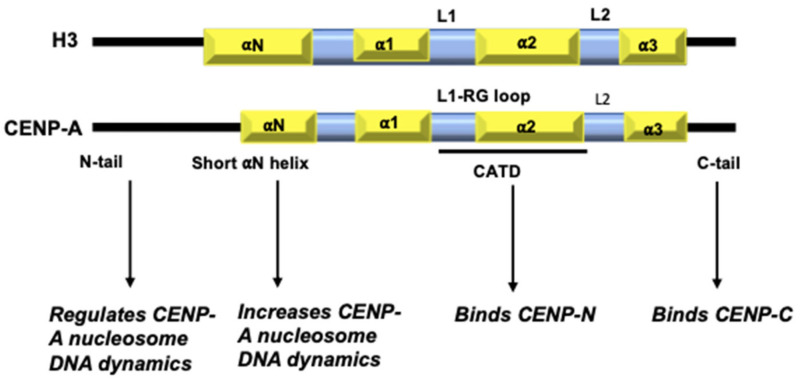
Domain organization of H3 and CENP-A: Key differences between H3 and CENP-A include: the sequence composition of CENP-A N-terminal tail; a one-turn shorter αN helix; differences in the HFD with a more exposed L1 and sequence composition of a short stretch at the C-terminal tail.

William Earnshaw serendipitously discovered CENP-A in 1985 in the sera of patients with CREST syndrome, and immunoblotting revealed that it is enriched at the centromeres [23,24]. Biochemical characterization of CENP-A showed that it co-purifies with histone proteins and is a bona fide element of the nucleosome core particles (NCP) [25,26]. It is constitutively associated with the centromere at all stages of the cell cycle and is the primary determinant of centromere identity [27]. CENP-A shares a ~60% homology with H3, mainly at the C-terminus, and replaces H3 in the centromeric nucleosomes [28]. Similar to H3, the human CENP-A has a disordered N-terminal tail, αN helix, the highly conserved histone fold made up of three α-helices, and a short C-terminal tail (Figure 1). The highly conserved Histone Fold Domain (HFD) of CENP-A differs from that of H3 and other histones. Specifically, the CENP-A HFD encompasses a unique CENP-A Targeting Domain (CATD) in the Loop1, helix 2 region (L1 α2) that is necessary and sufficient for centromere targeting. This is supported by ‘domain-swap’ experiments wherein when HFD of CENP-A and H3 are exchanged, H3 is targeted to the centromere but CENP-A is not. Further, the synthetic lethality caused by CENP-A depletion, could be overcome by the H3-HFD^CATD^ mutant [29]. 

During DNA replication, the CENP-A molecules are equally distributed between the sister chromatids, resulting in voids at places where CENP-A was originally present. These gaps are filled temporarily by the H3.3 ‘placeholder’ variant [12] and are quickly exchanged with CENP-A in a replication-independent manner in the early G1 phase of the cell cycle with the assistance of its chaperone [30]. The histone chaperone, identified as HJURP [31,32], specifically recognizes the CATD for targeting CENP-A at the centromere [33]. After being deposited, CENP-A recruits the essential 16-subunit Constitutive Centromere-Associated Network (CCAN) via direct interaction with CENP-C and CENP-N [34,35]. The CCAN is made up of multiple proteins such as CENP-C, CENP-N-L, CENP-H-I-K-M, CENP-T-W-S-X, and CENP-O-P-Q-R-U [36]. It further recruits components of the outer KT for microtubule (MT) attachment, thus forming a segregation-competent KT [37]. Therefore, CENP-A is the most important factor that defines and propagates the centromere identity. Without the incorporation of CENP-A, the identity of the centromere is lost which leads to chromosome segregation and kinetochore assembly defects [38].

Thus, it is the CATD in CENP-A that makes it possible for CCAN or HJURP to specifically recognize CENP-A over H3. The deposition of CENP-A has to be at the ‘right place and at the right time to ensure faithful chromosome segregation. A number of spaciotemporal factors ensure the rightful deposition of CENP-A to define the centromere and this process is called ‘centromere licensing’ [30]. For instance, pre-existing CENP-A is required at the centromere to recruit new CENP-A molecules to propagate the centromere identity during cell division [39]. Additionally, phosphorylation of the HJURP recruiter Mis18 complex by the Plk-1 kinase aids in its association with CENP-C at the centromere which acts as a mark for HJURP-assisted CENP-A deposition [40]. It should be noted here that how CENP-C specifically recognizes the centromere is not fully understood yet.

## 4. CENP-A Nucleosome

**Figure 2:**
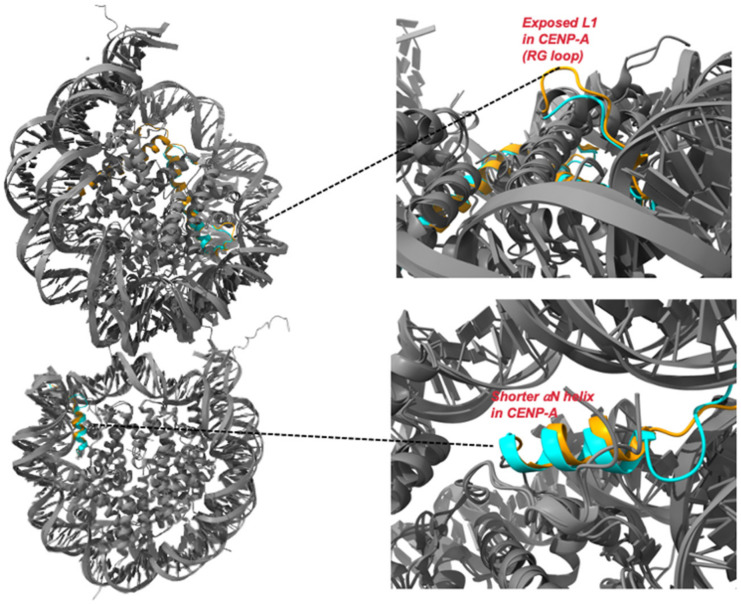
Comparison of crystal structures of H3 nucleosome and CENP-A nucleosome: Superimposition of crystal structures of H3 (PDB ID: 1AOI) and CENP-A (PDB ID: 6SE0) nucleosome. The two primary structural variations between H3 and CENP-A are depicted in the insets (**top** and **bottom**).

Crystal and Cryo-EM structures of the human CENP-A nucleosome [41,42] revealed that while the DNA is wrapped in a left-handed fashion just like in the canonical nucleosomes and the CENP-A-CENP-A interface compactness is comparable to that of the H3-H3 interface, there are some major variations:

(a) CENP-A αN helix is one-turn shorter compared to that of H3 (Figure 2) at the nucleosome entry and exit sites; as a result, there are fewer DNA interactions: ~13 bp DNA segment is detached from the histone surface at both ends [41]. This leads to an increase in the flexibility of the DNA ends and this phenomenon is independent of the DNA sequence [41,42,43]. Additionally, it has been shown that the N-terminal tail of CENP-A, but not H3, promotes this DNA unwrapping [42]. CENP-A nucleosomes wrap only about ~120 bp of DNA [41,42] as compared to the 147 bp wrapped by the canonical nucleosomes [43]. This observation is further supported by the crystal structure of a heterotypic nucleosome reconstituted using H3.3 and CENP-A in which the DNA at the CENP-A side has much greater flexibility, while DNA is firmly wrapped on the H3 side [44]. Increased dynamics of the DNA ends have been observed for CENP-A nucleosome in vivo in ChIP-seq experiments [45]. Thus, the presence of CENP-A leads to an alteration in the DNA dynamics in the nucleosome, which results in unique centromeric chromatin structures. For instance, studies on nucleosomes assembled on longer DNA demonstrate that the flexible DNA at the entry/exit locations causes an alternative path for the linker DNA that does not pass over the dyad. Hence, the CENP-A nucleosome is incompatible with linker histone H1 binding [46,47]. In fact, the exclusion of H1 may be required to promote binding with the CCAN components to mediate KT assembly. This claim is supported by studies that show that deletion of the flexible DNA ends in CENP-A nucleosome impairs CCAN binding [47,48]. Interestingly, mechanical measurements have recently demonstrated that the CENP-A nucleosomes are more elastic than H3 nucleosomes [49]. Further, high-speed Atomic Force Microscopy (AFM) showed that the frequency of local DNA unwrapping events is higher in CENP-A nucleosomes as compared to canonical ones [50]. Thus, we can speculate that increased DNA flexibility may lead to increased core dynamics in the CENP-A nucleosomes. The increased flexibility of DNA in the CENP-A nucleosome also results in an increased structural heterogeneity [41]. It is known that structural heterogeneity aids in binding to numerous proteins to mediate complex signalling and multiprotein assemblies [51]. While it has been shown that this flexibility is required for centromere function [47], much is still unknown and is a topic of research.
(b) CENP-A CATD L1 has extra residues called RG loop than that of H3 and protrudes out of the nucleosomes (Figure 2) and aids in the recruitment of CENP-N [33,52,53] as well as CENP-C according to a study in human cells [11]. CENP-N is essential for the recruitment of other CCAN components as mutations or depletion of CENP-N results in decreased binding with CENP-A which leads to defects in centromere assembly. Reduced CENP-A incorporation as well as reduced recruitment of CENP-H, CENP-I, and CENP-K has been observed [54]. Most recently, in vitro and in vivo experiments on CENP-A nucleosome arrays have demonstrated that CENP-N promotes the compaction of centromeric chromatin in an H1-like manner [51] to create a unique higher-order structure that may serve as an interaction platform for various centromere-specific proteins [55].
(c) Additionally, CENP-C, a highly conserved CCAN component and the central player that promotes and stabilizes the KT on the centromere, also forms contacts with the CENP-A C-terminal tail residues KDQ [56] via its C-terminus and the outer KT (MT-proximal end) KMN network via its N-terminus to establish a segregation-competent KT [57]. Cryo-EM structures of CENP-A in complex with CENP-C revealed that CENP-C promotes DNA unwrapping on binding to CENP-A nucleosomes via the H2A C-terminal tail and also alters the H4 tail conformation [57], possibly to assist in H4K20me1 (monomethylation), a mark that is crucial for KT assembly [58].

Crystal structures of the CENP-A nucleosomes with CENP-C and CENP-N (the core centromeric nucleosome complex (CCCN)) revealed that both proteins can co-exist on the nucleosome. In fact, both interact with the H4 tail to encourage H4K20me1 [58,59]. While CENP-C increases the DNA dynamics, subsequent binding of CENP-N reduces it [58], and this modulation of the DNA may be required for KT assembly. Further, these associations are dynamic throughout the cell-cycle [60], and we speculate that these may change the DNA dynamics on the CENP-A nucleosome to modulate various interactions.

(d) Specific hydrogen bonds develop between CENP-A protomers (mediated by the CATD) in the CENP-A nucleosome, causing structural aberrations around the CENP-A–CENP-A interface when compared to the H3-H3 interface [41], which, we believe, may have an impact on the centromeric nucleosome dynamics.
(e) As mentioned earlier, at the entrance and exit of the CENP-A nucleosome, a ~13 bp DNA segment is detached from the histone surface, giving CENP-A nucleosomes a more ‘open conformation’ compared to that of the canonical one. It is proposed that the unbound DNA constitutes a CENP-B box [61]. It is reported that CENP-B is required for the de novo assembly of CENP-A and other factors on the α-satellite DNA [62]. However, it appears that CENP-B function at the centromere is dispensable because CENP-B knockout mice are viable [63], and neocentromeres are devoid of CENP-B [7], questioning the importance of the centromeric DNA. However, since most studies involve engineered DNA sequences for in vitro nucleosome stabilization, it may result in the loss of important details about the nucleosome in its native DNA context. To summarize, the presence of CENP-A in the centromeric nucleosome (a) alters the core dynamics to create a unique chromatin structure at the centromere (b) serves as a platform for CCAN recruitment and subsequent KT assembly.

## 5. Post-Translational Modifications (PTMs) on CENP-A

**Figure 3:**
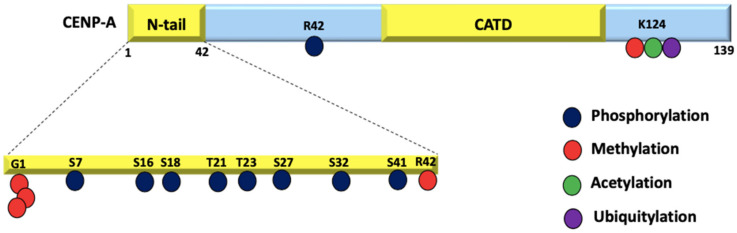
PTMs on CENP-A: CENP-A residues undergo phosphorylation, methylation, acetylation, and ubiquitylation. Due to the fact that the initial M cleavage was not taken into account in the original research, these residue positions have been moved up by 1.

CENP-A is decorated with a distinct set of PTMs compared to H3, which are crucial for centromere function [64]. Differential CENP-A PTMs are another important factor that define the centromere identity. Several PTMs, mainly methylation, phosphorylation, ubiquitylation, and acetylation have been identified for CENP-A that regulate its deposition at the centromere, its turnover rate as well as recruitment of the CCAN complex to the centromere to mediate kinetochore assembly [65] (Figure 3).

CENP-A has fewer modifications when compared to H3 due to significant sequence variability in its N-tail. With the absence of lysines, CENP-A is refractory to the activating and repressing marks (H3K4 and H3K27 methylation) [66]. In fact, the sequence variability of CENP-A promotes specific modifications that are relevant in the context of centromere function. For instance, the amino-terminal glycine trimethylation by NRMT1 is unique to CENP-A and is essential for proper chromosome segregation during mitosis [67]. Biochemical studies reveal that cells lacking the trimethylated CENP-A undergo chromosome missegregation as well as reduced levels of CENP-T and CENP-I expression at the centromere which leads to spindle multipolarity. Unmodified CENP-A when expressed in p53 -/- null cells result in rapid cell proliferation and early commencement of tumor in nude mice suggesting its possible role in cancer [67].

Molecular dynamics simulations on octameric CENP-A nucleosomes show that CENP-A K124 acetylation tightens the histone core and reduces the C-terminus accessibility leading to diminished CENP-C binding. This observation is corroborated by in vivo studies using a CENP-A K124Q mutant to mimic K124 acetylation. Further, a slight increase in mitotic errors is also observed [68]. In vivo biochemical studies on HeLa cells have demonstrated that the ubiquitylation of K124 promotes HJURP binding and subsequent localization to the centromere [69]. The function of K124 monomethylation remains unexplored. Lys-124 undergoes various modifications at different stages of the cell cycle. At mitotic departure and entry into the G1 phase, K124 is deubiquitylated and acetylation is enriched, followed by deacetylation and further monomethylation during the G1/S phase [70,71,72].

The crystal structure of HJURP-CENP-A-H4 shows that HJURP binds to CENP-A via the CATD, and this interaction is the primary determinant of CENP-A targeting the centromere [33]. However, phosphorylation of CENP-A residues has been reported to play a role in its centromeric deposition as well. Biochemical studies have shown that S18 and S68 phosphorylation negatively regulates CENP-A deposition by hindering HJURP binding [11,73,74]. Phosphorylated Ser-16 and -18 have been postulated to form salt bridges with the nearby Arg residues. The presence of salt bridges alters the organization of CENP-A nucleosomal arrays in vitro [69], suggesting a potential role of PTMs in altering higher-order chromatin structures. Various contradicting studies on the S7 phosphorylation of CENP-A have led to non-conclusive interpretations about its role in centromere assembly and function [75,76,77]. A recent study has addressed these inconsistencies by using state-of-the-art genome editing tools to conclude that S7 phosphorylation is dispensable for centromere function. The authors suggest that this low-abundance modification may have a not-yet-known biological function such as in the maintenance of sister chromatid cohesion [78]. While ubiquitination plays a role in regulating CENP-A levels, no E3 ligases have been identified so far. Multiple E3 ligases such as Psh1, Rcy1, Ubr1, and Slx5 independently regulate the levels of Cse4 in yeast cells [79]. Rcy1, Ubr1, and Slx5 have known homologs in humans, providing a hint towards CENP-A protein regulation in a similar manner to that of the budding yeast. Evidently, more research is required to identify and study the PTMs that decorate CENP-A and how they specify the centromere identity.

## 6. H2A.Z

Studies on the inactive X chromosome centromere as a model system have identified that the H2A variant- H2A.Z is prevalent across the centromere in association with the dimethyl K4 H3 and trimethyl K9 H3 marks. [80]. Disruption of H2A.Z causes a loss of HP1 binding even in the presence of the H3K9 trimethylation mark, asserting the importance of H2A.Z for heterochromatinization [81]. In fact, in vitro reconstructed H2A.Z containing nucleosome arrays generate a condensed chromatin state in comparison to H2A. Hence, it has been proposed that H2A.Z maintains the integrity of the heterochromatin at the centromere which is crucial for sister chromatid interactions [82]. This is supported by the observation that loss of centromeric H2A.Z leads to loss of gene silencing and chromosome segregation defects in fission yeast [83]. The complex spatial distribution of H2A.Z and the PTM marks in the centro- and pericentro-chromatin provides a unique conformation to the centromere to facilitate its functions. However, extensive studies are required to study the mechanism by which this sophisticated spatial organization complements the centromere function.

## 7. CENP-A and Human Diseases

Defective centromere function and kinetochore assembly are the known causative agents of aneuploidy, which leads to genomic instability which is a hallmark of cancer [13]. CENP-A is one of the key molecular factors involved in centromere maintenance and kinetochore assembly and hence its deregulation is a major contributor toward defective cell division [84]. In fact, when CENP-A^CID^ was overexpressed in Drosophila, it was found to mis-localize at non-centromeric locations. Chromosome mis-segregation, aneuploidy, and developmental abnormalities were all brought on by CID mis-localization, which led to the development of ectopic centromeres and multicentric chromosomes [85]. Furthermore, a high expression level of the CENP-A mRNA has been observed in hepatocellular carcinoma (HCC) patients [86]. Similarly, transcriptional overexpression of CENP-A was also seen in colorectal cancer tissues. Moreover, CENP-A was discovered to be overexpressed in neoplasic intratubullar germ cells along with other core markers, leading to the proposal of the centromere antigen as a novel biological marker of human disease [87].

## 8. Conclusions

In higher eukaryotes such as humans, the centromere identity is independent of the DNA sequence. It is well established that the nucleosomes containing CENP-A generate a unique higher-order chromatin structure that marks the centromere on the chromatin. Additional factors include the epigenetic reprogramming of chromatin in the terms of differential PTMs on CENP-A, the H2A.Z variant, and other centromeric proteins such as CENP-C. While significant progress has been made in the past decade to highlight how PTMs and H2A.Z shape up the centromeric chromatin, extensive studies are still required to understand the underlying mechanisms that enable them to do so. Finally, CENP-A can also be an attractive drug target in cancer therapy as its deregulation has been linked to both colorectal and hepatocellular cancers.

## Data Availability

Not applicable.
